# Dexamethasone Intravitreal Implant (Ozurdex) for Long-Term Macular Edema after Epiretinal Membrane Peeling Surgery

**DOI:** 10.1155/2018/5832186

**Published:** 2018-12-10

**Authors:** Yo-Chen Chang, Pei-Kang Liu, Tzu-En Kao, Horng-Jiun Wu, Kai-Chun Cheng, Kuo-Jen Chen, Kwou-Yeung Wu, Wen-Chuan Wu

**Affiliations:** ^1^Department of Ophthalmology, Kaohsiung Medical University Hospital, Kaohsiung 80708, Taiwan; ^2^Department of Ophthalmology, School of Medicine, Kaohsiung Medical University, Kaohsiung 80708, Taiwan; ^3^Department of Ophthalmology, Kaohsiung Municipal Ta-Tung Hospital, Kaohsiung Medical University, Kaohsiung, Taiwan; ^4^Department of Ophthalmology, Kaohsiung Municipal Hsiao-Kang Hospital, Kaohsiung Medical University, Kaohsiung 81267, Taiwan

## Abstract

**Purpose:**

To investigate the functional and anatomical outcome of the 0.7 mg dexamethasone (Ozurdex) intravitreal implant (IVD) in eyes with long-term macular edema after macular epiretinal membrane removal.

**Methods:**

We enrolled 40 eyes with persistent macular edema at least 12 months after epiretinal membrane removal. Twenty eyes in the IVD group received IVD and the other 20 eyes were in the control group. The main outcome measures were change in best-corrected visual acuity (BCVA) and central foveal thickness (CFT).

**Results:**

For eyes in the IVD group, the mean BCVA improved by 3.45 lines to 0.47 logMAR one month after IVD. However, the mean BCVA improved by only 0.14 lines to 0.74 logMAR at the same time in eyes in the control group. Six months later, the mean BCVA improved to 0.31 and 0.74 logMAR in the IVD and control groups, respectively. In the IVD group, the mean CFT decreased rapidly by 116.8 *μ*m to 333.9 *μ*m one month after IVD. Thereafter the CFT decreased at a slower pace. In the control group, the CFT remained static during the follow-up period. However, in the IVD group, 6 months after IVD, the CFT seemed to have a tendency to increase.

**Conclusions:**

Single IVD could significantly decrease macular edema and improve visual outcome for eyes with persistent long-term macular edema after macular ERM removal and the effect can be sustained as long as 6 months after the initial injection. However, in order to maintain the visual and anatomical outcome, repeat IVD might be considered if macular edema recurs.

## 1. Introduction

Idiopathic epiretinal membrane (ERM) is a degenerative disorder occurring in the vitreomacular interface that usually affects the central vision in the affected eye [1]. The manifestation of an ERM can range from asymptomatic to photopsia, metamorphopsia, macropsia or micropsia, decreased visual acuity (VA), and even central vision loss. In 1978, Dr. Machemer first described pars plana vitrectomy (PPV) with membrane peeling for ERM. Since then this surgery has become a well-established method for the removal of ERM with good results [2]. Surgical removal of the membrane in patients with significant symptoms can improve visual acuity and reduce metamorphopsia in approximately 70% of cases [3–5]. However, persistent residual intraretinal edema is sometimes still present, limiting the possibilities of complete visual function recovery [6, 7]. From our previous study, stable vision is usually achieved approximately 9 months after PPV, and intravitreal injection of bevacizumab cannot further improve the final visual and anatomical outcome [8]. Therefore, vascular endothelial growth factor (VEGF) might play a minor role in the pathogenesis of persistent macular edema after ERM peeling. In addition to VEGF, inflammatory trauma by mechanical membrane peeling might be associated with postoperative macular edema [9, 10]. To further reduce macular edema, intravitreal injection of triamcinolone acetonide (IVTA) after the removal of the ERM has been shown to facilitate fluid absorption and may speed up improvement of the anatomic and functional outcome in short-term follow-up [11]. The possible reason for the short-term effect of intraoperative IVTA might be explained by the relatively rapid clearance in vitrectomized eyes, which may abate its effect for macular edema [12, 13]. Therefore, there may be a rationale for using a sustained-release device to improve this condition. The dexamethasone intravitreal implant (IVD) (Ozurdex; Allergan, Irvine, CA) provides a slow release and stable dose of steroid [14]. IVD has been widely used for treating noninfectious posterior uveitis, macular edema secondary to retinal vascular occlusion (RVO), diabetic macular edema (DME), and cystoid ME (CME) after cataract surgery [15–17]. Therefore, in our present study, we tried to determine the efficacy of the 0.7 mg dexamethasone intravitreal implant to treat long-term persistent macular edema (no tendency to resolve at least 12 months after surgery) after removal of idiopathic ERM.

## 2. Patients and Methods

This is a retrospective, nonrandomized study including patients who were diagnosed with idiopathic ERM between January 2014 and June 2015. This study followed the tenets of the Declaration of Helsinki in 1964. All operations were performed by the same physician (W. C. W.) who is a retinal specialist experienced in vitreoretinal and cataract surgery as well as intravitreal injection. The surgical procedure and postoperative medication were using the methods as described previously [8]. All patients previously underwent standard three-port transconjunctival 25-gauge PPV assisted with triamcinolone for membrane peeling and indocyanine green (ICG) for internal limiting membrane (ILM) peeling using high-magnification viewing lens and intraocular forceps. PVD was induced with active suction of ocutome over the optic disc if the PVD was not already present. Concomitant cataract surgery was performed on phakic patients. At the end of surgery, all patients received a subconjunctival injection of 2 mg Rinderon (betamethasone 4 mg/mL). After surgery, Tobradex eye drops (0.3% tobramycin and 0.1% dexamethasone) 4 times daily, Acular eye drops (ketorolac 0.4%) 3 times daily, and Mydriacyl (0.5% tropicamide) 3 times daily were administered to all patients for 2 weeks. Then, the Tobradex eye drops were switched with Sinomin eye drops (4% sulfamethoxazole) and 0.1% fluorometholone 4 times daily during the follow-up period. In addition, Acular eye drops (ketorolac 0.4%) three times daily were prescribed for patients who had macular edema during the follow-up period. There were no significant complications such as rupture of posterior capsule, retinal detachment, iatrogenic macular hole, postoperative vitreous hemorrhage, or endophthalmitis that affected visual outcome during or after surgery.

At least twelve months after PPV, patients who met the following criteria were selected for intravitreal injection of dexamethasone (IVD) treatment: (1) presence of macular edema ≥300 *μ*m as detected by optical coherence tomography (OCT) and (2) best-corrected visual acuity (BCVA) of 20/40 or less after surgery [8].

Patients with preexisting ocular diseases (i.e., chronic inflammatory or neoplastic disorders, retinal vascular occlusion glaucoma, or high myopia) were excluded, as well as those with systemic diseases (uncontrolled hypertension or diabetes).

After a complete discussion of the benefits, risks, and alternative treatment, the decision to treat with IVD was made by the eligible patients themselves. If the patient decided to proceed with IVD, a consent form had to be signed by the patient before treatment. In the IVD group, each patient received an intravitreal injection of the 0.7 mg dexamethasone intravitreal implant at least 12 months after PPV. Postoperatively, gentamycin eye drops were administered 4 times daily for 1 week. Meanwhile, those eligible patients who refused IVD were enrolled in the control group.

In order to compare the parameters between the IVD group and the control group, we defined “baseline” as the time point of (1) receiving IVD that was at least 12 months after PPV for patients in the IVD group and (2) the patient deciding not to receive IVD (at least 12 months after PPV for patients in the control group). The treated patients in the IVD group were seen at 1 week after baseline for check-up of injection wound and intraocular pressure. To evaluate the effects and safety of treatment, all recruited patients in both groups underwent comprehensive ophthalmic examinations including visual acuity by Snellen charts, slit-lamp biomicroscopy, Goldmann applanation tonometry, and ophthalmoscopy at 1, 2, 3, and 6 months after baseline. Measurements of macular thickness were performed using the spectral-domain-OCT (SD-OCT [Heidelberg Retina Angiograph 2, Heidelberg Engineering, Heidelberg, Germany]). In all measurements, the central foveal thickness (CFT) was assessed within a 1 mm diameter circle in the central macula. For better comparison of visual acuity between the groups, the visual acuity by Snellen chart was converted to the logarithm of the minimum angle of resolution (logMAR) at baseline and each follow-up visit.

### 2.1. Statistical Analysis

All data were statistically analyzed by Student's *t*-test or the *χ*^2^ test using SPSS statistical software (version 24.0; SPSS Inc., Chicago, Ill., USA). A *p* value of <0.05 was considered statistically significant.

## 3. Results

### 3.1. Baseline Demographic Data

During the period of one and half years, we reviewed a total of 400 eyes of 360 patients with idiopathic ERM who underwent PPV and ERM peeling (Table 1). There were 40 eyes of 40 patients who met the inclusion criteria. Table 1 shows the baseline demographics of these 40 patients. Both the IVD group and the control group consisted of twenty eyes. Patients in both groups were followed-up for at least 12 months after baseline. The mean interval from PPV to IVD was 58.8 ± 4.0 weeks (range: 53 to 66 weeks) in the IVD group and the mean interval from PPV to baseline was 57.7 ± 4.0 weeks (range: 52 to 66 weeks). The mean BCVA before PPV was 0.81 ± 0.30 in the IVD group and 0.75 ± 0.38 logMAR in the control group. Combined phacoemulsification with intraocular lens implantation, PPV, and membrane peeling were performed for 8 eyes in the IVD group and 10 eyes in the control group. The remaining 12 pseudophakic eyes in the IVD group and 10 pseudophakic eyes in the control group received PPV and membrane peeling only. At baseline, the SD-OCT measured mean CFT was 450.7 ± 72.4 *μ*m in the IVD group and 466.2 ± 82.8 *μ*m in the control group.

### 3.2. Temporal Change of CFT

**Figure 1:**
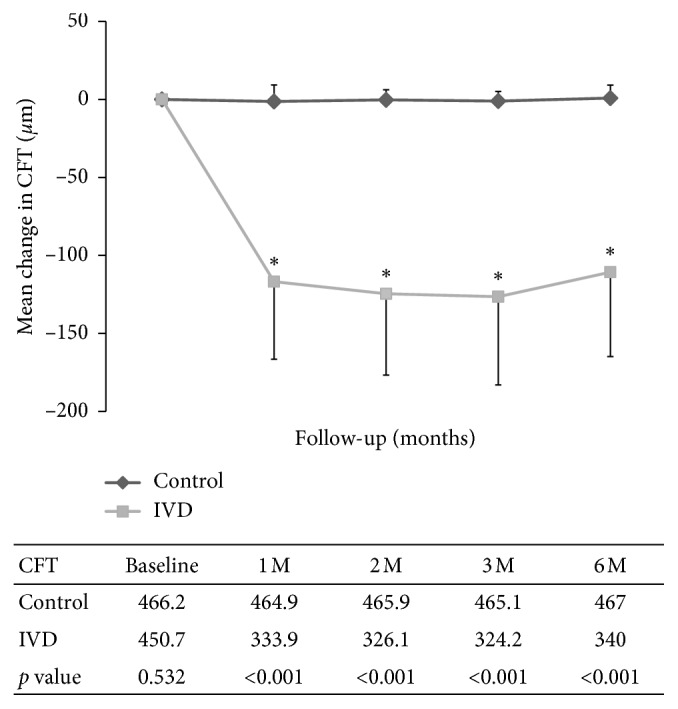
Temporal change of central foveal thickness (CFT) after IVD. Compared to baseline, the mean CFT at 1 month after IVD decreased rapidly by 116.8 ± 49.7 to 333.9 ± 50.9 *μ*m for the treatment group and by only −1.3 ± 10.6 to 464.9 ± 76.8 *μ*m for the control group (*p* < 0.01 between the IVD and control groups). Thereafter, the CFT in the IVD group decreased at a slower rate. The CFT in the control group remained static during the follow-up period. At 6 months, the mean CFT decreased by 110.7 ± 54.1 to 340.0 ± 53.1 *μ*m for the treatment group and by only 0.85 ± 8.2 to 467.0 ± 79.8 *μ*m for the control group (*p* < 0.01 between the IVD and control groups). ^*∗*^*p* < 0.01 compared to baseline.

All patients in the IVD group received single IVD. Compared to baseline, the mean CFT at 1 month after IVD decreased rapidly by 116.8 ± 49.7 to 333.9 ± 50.9 *μ*m for the IVD group and by only −1.3 ± 10.6 to 464.9 ± 76.8 *μ*m for the control group (*p* < 0.01 between the IVD and control groups) (Figure 1). Thereafter, the CFT in the IVD group decreased at a slower rate. The CFT in the control group remained static during the follow-up period. At 6 months, the mean CFT decreased by 110.7 ± 54.1 to 340.0 ± 53.1 *μ*m for the IVD group and by only 0.85 ± 8.2 to 467.0 ± 79.8 *μ*m for the control group (*p* < 0.01 between the IVD and control groups). The mean reduction in CFT differed significantly between the IVD and control groups at 1, 2, 3, and 6 months after baseline (*p* < 0.01). However, 6 months after baseline, the CFT in the IVD group seemed to have a tendency to increase. For eyes that received combined surgery (8 eyes in the IVD group and 10 eyes in the control group), the mean CFT in the IVD group improved from 442.3 ± 40.6 *μ*m at baseline to 343.0 ± 59.2 *μ*m at 6 months (*p* < 0.001). However, the mean CFT in the control group was 478.2 ± 97.8 at baseline and 480.5 ± 93.0 at 6 months (*p*=0.52). Preoperative pseudophakic eyes that received PPV and membrane peeling (12 eyes in the IVD group and 10 eyes in the control group), the mean CFT in the IVD group improved from 456.1 ± 89.0 *μ*m at baseline to 337.9 ± 51.2 *μ*m at 6 months (*p* < 0.001). However, the mean CFT in the control group was 454.1 ± 67.8 at baseline and 453.5 ± 66.2 at 6 months (*p*=0.70).

### 3.3. Temporal Change of Visual Acuity

**Figure 2:**
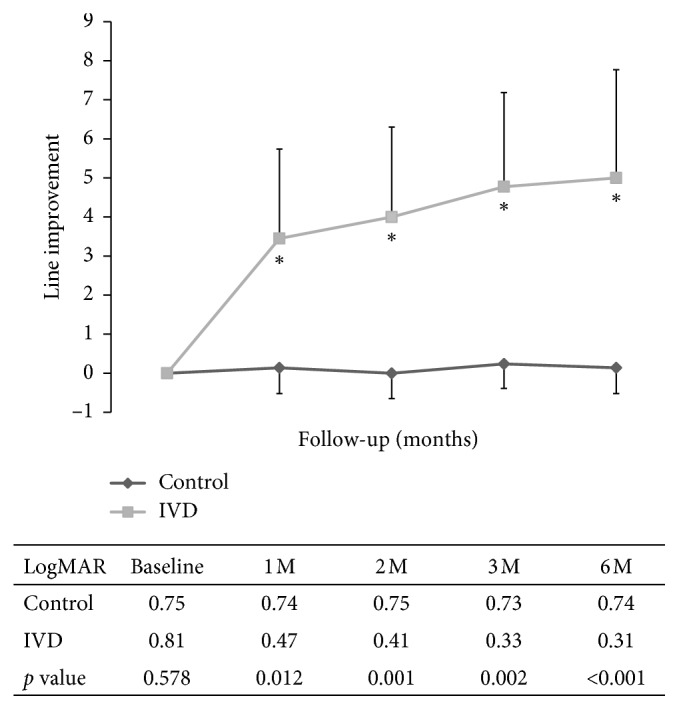
Temporal change of BCVA by line and mean BCVA after IVD. At 1 month after IVD, the mean BCVA had improved dramatically by 3.45 ± 2.29 and only 0.14 ± 0.66 lines from baseline in the IVD and control groups, respectively (*p* < 0.01, IVD vs. control). Thereafter, the BCVA in the IVD group continued to improve at a slower rate compared to stable BCVA in the control group. At 6 months after IVD, the mean BCVA had increased by 5.0 ± 2.77 lines to 0.31 ± 0.22 logMAR and only 0.14 ± 0.66 lines to 0.74 ± 0.36 logMAR from baseline in the IVD and control groups, respectively (*p* < 0.01). The logMAR BCVAs and line improvement differed significantly at 1, 2, 3, and 6 months after treatment between the two groups. ^*∗*^*p* < 0.05 compared to baseline.

At baseline, the mean BCVA was 0.81 ± 0.30 and 0.75 ± 0.38 logMAR in the IVD and control groups, respectively (*p*=0.58) (Figure 2). In the IVD group, the BCVA improved significantly after baseline. At 1 month, the mean BCVA had increased dramatically by 3.45 ± 2.29 and only 0.14 ± 0.66 lines from baseline in the IVD and control groups, respectively (*p* < 0.001). Thereafter, the BCVA in the IVD group continued to improve at a slower rate compared to stable BCVA in the control group. At 6 months, the mean BCVA had increased by 5.0 ± 2.77 lines to 0.31 ± 0.22 logMAR and only 0.14 ± 0.66 lines to 0.74 ± 0.36 logMAR from baseline in the treatment and control groups, respectively (*p* < 0.001). After baseline, the logMAR BCVAs and line improvement were significantly better in the IVD group than in the control group and differed significantly at each follow-up. For eyes that received combined surgery (8 eyes in the IVD group and 10 eyes in the control group), the mean BCVA in the IVD group improved from 0.74 ± 0.27 logMAR at baseline to 0.23 ± 0.22 logMAR at 6 months (*p*=0.002). However, the mean BCVA in the control group was 0.70 ± 0.27 at baseline and 0.68 ± 0.24 at 6 months (*p*=0.44). For preoperative pseudophakic eyes that received PPV and membrane peeling (12 eyes in the IVD group and 10 eyes in the control group), the mean BCVA in the IVD group improved from 0.86 ± 0.31 logMAR at baseline to 0.36 ± 0.22 logMAR at 6 months (*p* < 0.001). However, the mean BCVA in the control group was 0.80 ± 0.47 at baseline and 0.79 ± 0.46 at 6 months (*p*=0.65).

### 3.4. Intraocular Pressure

In the IVD group, three patients (15%) experienced IOP elevations of 10 mm Hg or more from baseline at 1 month postoperatively. These three patients receiving single topical anti-glaucoma medication and no IOP elevation were noted at the 2-, 3-, or 6-month follow-up visits. No new IOP-lowering medication was required in any patients at the time of the latest follow-up. In the control group, none of the patients experienced IOP elevations of 10 mm Hg or more from baseline at any follow-up visit.

### 3.5. Case Presentation

**Figure 3:**
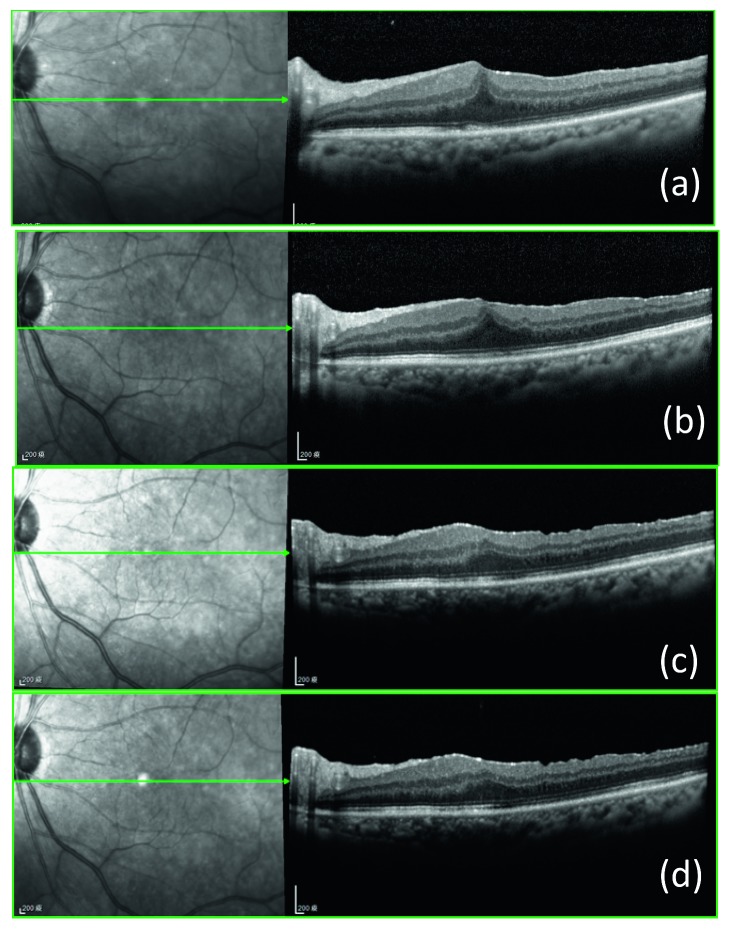
Pre- and postoperative OCT findings in a patient with long-term persistent macular edema after membrane peeling and internal limiting membrane peeling. Preoperative image shows an ERM overlying the macula. The CFT and BCVA were 436 *μ*m and 20/100, respectively (a). Thirteen months after operation, no ERM or ILM was noted but the BCVA was still 20/100 with the CFT of 449 *μ*m (b). At one month after IVD, the CFT decreased to 350 *μ*m and the BCVA improved to 20/30 (c). At three months after IVD, the CFT decreased to 290 *μ*m and the BCVA improved to 20/22 (d). Six months after IVD, the CFT was 306 *μ*m and the BCVA maintained at 20/22 (e).

**Figure 4:**
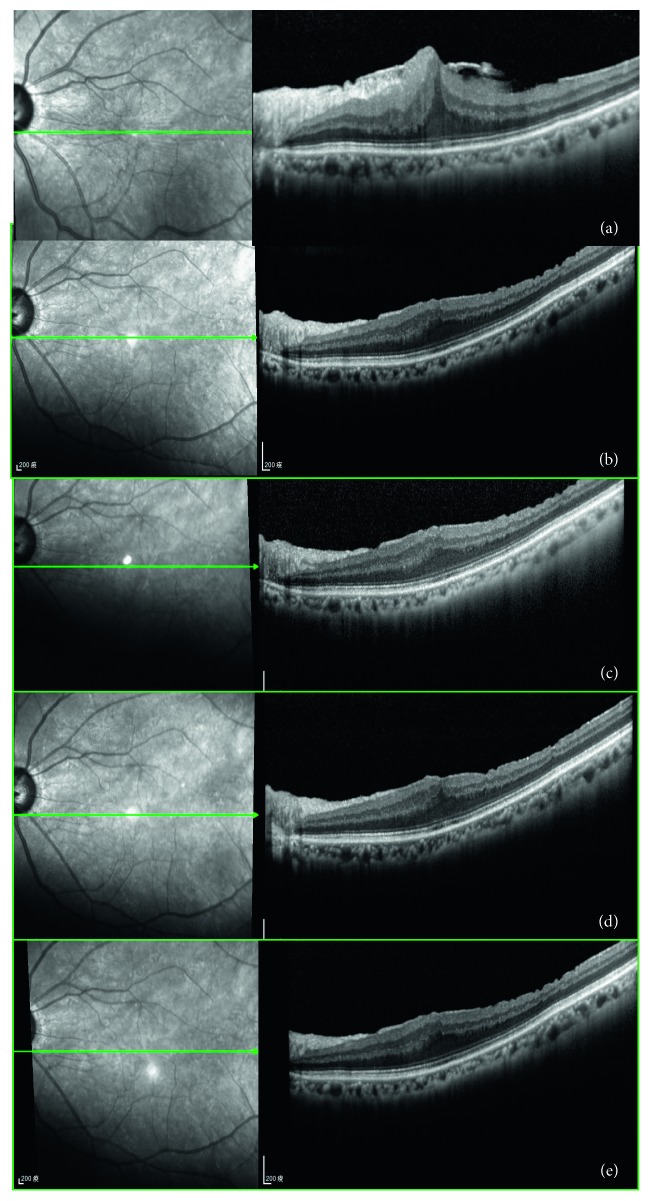
Pre- and postoperative OCT findings in a patient with more long-term persistent macular edema after membrane peeling and internal limiting membrane peeling. The preoperative OCT image showed prominent ERM overlying the macular surface, the CFT was 656 *μ*m and the BCVA was 20/400 (a). Thirty-six months after operation, no ERM was noted, but the BCVA had only improved to 20/200 with the CFT of 422 *μ*m (b). One month after IVD, the CFT decreased to 407 *μ*m and the BCVA improved to 20/22 (c). Three months after IVD, the CFT slightly decreased to 398 *μ*m and the BCVA maintained at 20/22 (d). Six months after IVD, the CFT was 385 *μ*m and the BCVA further improved to 20/20 (e).

Figure 3 represents OCT images of the pre- and postoperative course of the eye that suffered from long-term persistent macular edema after ERM and ILM peeling. The preoperative OCT image showed prominent ERM overlying the macular surface, the CFT was 436 *μ*m and the BCVA was 20/100 (Figure 3(a)). Thirteen months after operation, no ERM was noted, but the BCVA was still 20/100 with the CFT of 449 *μ*m (Figure 3(b)). One month after IVD, the CFT decreased to 350 *μ*m and the BCVA improved to 20/30 (Figure 3(c)). Three months after IVD, the CFT further decreased to 290 *μ*m and the BCVA improved to 20/22 (Figure 3(d)). Six months after IVD, the CFT was 306 *μ*m and the BCVA maintained at 20/22 (Figure 3(e)).  Figure 4 represents OCT images of the pre- and postoperative course of the eye that suffered from more long-term persistent macular edema after ERM and ILM peeling. The preoperative OCT image showed prominent ERM overlying the macular surface, the CFT was 656 *μ*m and the BCVA was 20/400 (Figure 4(a)).Thirty-six months after operation, no ERM was noted, but the BCVA only improved to 20/200 with the CFT of 422 *μ*m (Figure 4(b)). One month after IVD, the CFT decreased to 407 *μ*m and the BCVA improved to 20/22 (Figure 4(c)). Three months after IVD, the CFT slightly decreased to 398 *μ*m and the BCVA maintained at 20/22 (Figure 4(d)). Six months after IVD, the CFT was 385 *μ*m and the BCVA further improved to 20/20 (Figure 4(e)).

## 4. Discussion

Pars plana vitrectomy with membrane peeling is a useful technique for patients with symptomatic ERM, and most patients will have a favorable outcome [2–6]. However, visual recovery may take several months to as long as 1 year as the retinal morphology normalizes slowly [8, 18]. However, persistent residual intraretinal edema is sometimes still present, limiting the possibility of a complete visual function recovery [6, 7]. To date, the mechanism of macular edema in idiopathic ERM has not been fully studied. Ahn et al. hypothesized that the thickening macula in eyes with ERM might be due to traction-induced distortion of the neurosensory retina or macular edema from breakdown of the blood-retinal barrier [19]. Mandelcorn et al. demonstrated that positive immunostain for VEGF and transforming growth factor-beta (TGF-beta2) were present in 85% of idiopathic ERM specimens [20]. However, in our previous study, for patients with persistent macular edema after ERM peeling, there were no significant differences of final visual and anatomical outcome between patients with or without bevacizumab injection [8]. Therefore, bevacizumab might somewhat reduce macular edema by acting as an anti-VEGF agent which is believed to play a minor role in the pathogenesis of postoperative macular thickening [11].

Harada et al. hypothesized that the release of inflammatory cytokines and growth factors may contribute to ERM proliferation as well as accompanying macular edema in many cases [21]. Vinores et al. suggested that postoperative macular edema is in part due to breakdown of the blood-retinal barrier, then release of inflammatory cytokines from preoperative mechanical traction, and intraoperative manipulation [22]. Corticosteroids are very potent anti-inflammatory agents that can block several pathological processes which are thought to be involved in the development of macular edema in several ways: inhibiting the synthesis of VEGF, prostaglandins, and many proinflammatory cytokines, reducing fibrin deposition, preventing leukocyte migration, and stabilizing endothelial cell tight junctions [23]. However, the type of corticosteroid and administrating route may affect the efficacy to specific diseases. Systemic corticosteroids might cause certain adverse events such as adrenal suppression, Cushingoid state, osteoporosis, and exacerbation of diabetes [24–27]. Topical or local administration usually leads to a suboptimal drug level in the vitreous and may be associated with relatively high systemic concentrations [28–30]. Therefore, direct intravitreal injection of corticosteroids seems to be the better way to achieve optimal drug level in the vitreous.

Dexamethasone is the most water-soluble and also a potent synthetic glucocorticoid. The anti-inflammatory and immunosuppressant activity of dexamethasone is 30 times more than cortisol [31] and 12.5 times more than triamcinolone [32]. In addition, the half-life of dexamethasone is the shortest among other steroids and is less likely to aggregate in the trabecular meshwork and therefore cause elevation of intraocular pressure [33].

The reason for corticosteroids being effective treatment for long-term persistent macular edema after ERM and ILM peeling in the present study remains unclear. The possible explanation may be due to the effects on Müller cells during ERM and ILM peeling. Müller cells, the major type of glial cells in the retina, are responsible for the homeostatic and metabolic support of retinal neurons. While their cell bodies are located in the inner nuclear layer of the retina, they span across the entire retina [34]. They have been further identified as fundamental to the transmission of light through the vertebrate retina due to their unique funnel shape [35]. Under certain conditions, a subset of Müller cells may differentiate to neural progenitor/stem cells which regenerate lost photoreceptors and neurons [36]. During ILM peeling, the Müller cell footplates which build up the outer portions of the ILM might suffer from mechanical damage [37]. The initial mechanical damage of the retinal surface might be associated with secondary biochemical pathways involving different cytokines and growth factors, which may contribute to the development of diffuse retinal thickening visible on SD-OCT. Therefore, corticosteroids may be able to antagonize the secondary inflammatory effects triggered by the mechanical distortion and thus accelerate the resolution of macular edema, helping the restoration of physiologic function of Müller cells and therefore improving visual function.

From literature review, there were several reports with inconsistent results regarding the effect of the 0.7 mg dexamethasone intravitreal implant of macular edema after ERM peeling surgery [38–42]. Furino et al. reported a single injection of the 0.7 mg dexamethasone intravitreal implant was effective in the treatment of at least 2 months duration of macular edema secondary to combined cataract extraction and vitrectomy for macular pucker removal allowing a stable visual acuity recovery in a small retrospective series of 8 eyes [38]. Taney et al. reported that four of five eyes showed reduction in macular thickness and visual acuity improved by one or more Snellen lines after dexamethasone intravitreal implant in a small retrospective series of 5 eyes suffering from persistent macular edema after vitrectomy for ERM [39]. However, both studies were limited by a relatively small sample size and lack of control group. Yonekawa et al. reported that both dexamethasone intravitreal implant and triamcinolone acetonide are effective in improving central macular thickness and visual acuity while the intraoperative dexamethasone intravitreal implant or triamcinolone acetonide is used after vitrectomy and membrane peeling [40]. However, recently, Guidi et al. reported that the intraoperative sustained-release dexamethasone implant did not result in a significant change in macular thickness and volume compared with the vitrectomy alone without dexamethasone implant at 6-month follow-up [42]. The possible explanation for their negative results may be due to the timing of intervention. According to our previous study, for patients with idiopathic ERM, stable vision is usually achieved approximately 9 months after PPV and membrane peeling [8]. Therefore, it is reasonable to hold the IVD until the macular thickening persists at least 9 months postoperatively.

In our present study, compared to patients in the control group, in patients with long-term persistent macular edema after ERM peeling surgery, IVD can both significantly reduce CFT and improve visual acuity after injection, and the effect can last as long as 6 months. There were three patients (15%) among the IVD group experiencing IOP elevations of 10 mm Hg or more from baseline at 1 month postoperatively. The IOP in these 3 patients can be well-controlled by single topical anti-glaucoma medication and no new IOP-lowering medication was required in any patients at the time of latest follow-up.

In summary, the present study showed a single injection of the 0.7 mg dexamethasone intravitreal implant may be effective to improve visual function of patients with long-term (longer than 12 months) persistent macular edema secondary to vitrectomy with membrane and ILM peeling for idiopathic ERM. However, there are some limitations of this study including its retrospective nature and relatively small number of patients. Therefore, these results need to be confirmed by a large prospective and randomized trial.

## Figures and Tables

**Table 1:** Patient demographics at baseline.

Feature	IVD group	Control group	*p* value
Patient number	20	20	1
Age (years)	63.9 ± 6.2	62.8 ± 5.8	0.57
Male : female	10 : 10	8 : 12	0.75
Interval from PPV to IVD or baseline (wks)	58.8 ± 4.0	57.7 ± 4.0	0.61
LogMAR BCVA	0.81 ± 0.30	0.75 ± 0.38	0.58
CFT (*μ*m)	450.7 ± 72.4	466.2 ± 82.8	0.53

BCVA: best-corrected visual acuity; CFT: central foveal thickness; IVD: dexamethasone intravitreal injection; logMAR: logarithm of the minimum angle of resolution.

## Data Availability

The data used to support the findings of this study are available from the corresponding author upon request.
